# Impact of *IL-6* and *IL-1β* Gene Variants on Non-small-cell Lung Cancer Risk in Egyptian Patients

**DOI:** 10.1007/s10528-023-10596-2

**Published:** 2023-12-16

**Authors:** Yomna F. Metwally, Afaf M. Elsaid, Rana R. Elsadda, Sherif Refaat, Rasha F. Zahran

**Affiliations:** 1 Biochemistry Department, Faculty of Science, Damietta University, Damietta, Egypt; 2 Genetics Unit, Children Hospital, Mansoura University, Mansoura, Egypt; 3 Oncology Department, Oncology Center, Mansoura University, Mansoura, Egypt

**Keywords:** Lung cancer, Gene polymorphism, Inflammatory cytokines, ARMS-PCR, Egypt

## Abstract

Lung cancer is a serious health and life issue, with the fastest-growing incidence and fatality rates worldwide. It is now clear that inflammation is a key factor involved in all aspects of carcinogenesis, notably lung cancer development. Genetic changes, including polymorphisms in inflammatory genes, are supposed to be a significant cause of increased lung cancer risk. The main idea of this research was to disclose the linkage between both *IL-6* rs1800795 and *IL-1β* rs16944 variants and susceptibility to non-small-cell lung cancer (NSCLC) in Egyptians. This case–control design was composed of 127 cases and 138 controls, which were genotyped using the ARMS-PCR technique. To examine the NSCLC susceptibility under various genetic models, the odds ratio (OR) and 95% confidence intervals (CIs) were determined by logistic regression. Rs1800795 of the *IL-6* gene was linked to higher odds of NSCLC under the allele model (adjusted, OR 2.28; 95% CI 1.2–4.33; *p* = 0.011). In the genetic models, *IL-6* rs1800795 elevated the odds of NSCLC, while *IL-1β* rs16944 decreased the odds of NSCLC. Stratification analysis showed that *IL-6* rs1800795 greatly increased the NSCLC risk in females and adenocarcinoma subtypes, whereas *IL-1β* rs16944 largely decreased the NSCLC risk for males, patients aged < 55, and nonsmokers. Regarding clinical data, the *IL-6* variant was remarkably correlated with tumor size. This work primarily established that *IL-6* and *IL-1β* variants have a great impact on NSCLC development in the Egyptian population; thus, it may be a supportive guide for earlier NSCLC prevention.

**Supplementary Information:**

The online version contains supplementary material available at 10.1007/s10528-023-10596-2.

## Introduction

Lung cancer stands as the most fatal malignant tumor, which poses a risky health and life conundrum worldwide. So far, lung cancer has the highest fatality rate and is the second-most frequently diagnosed cancer. In 2020, almost 1.8 million lung cancer deaths (18% of all cancer deaths) and approximately 2.2 million new cases (11.4% of all cancers) were found (Kaanane et al. 2022). In men, its rank is the first for incidence and mortality, whereas in women, its rank is the third for incidence, after breast and colorectal cancer, and the second for death, after breast cancer. The lung cancer prevalence is approximately two times higher in males than in females worldwide (Sung et al. 2021). In Egypt, lung cancer represents the fastest-growing tumor with a ratio of 3.2:1 male-to-female. By increasing the number of women smokers, the prevalence of female lung cancer is elevating (El-Moselhy and Elrifai 2018).

Small-cell lung carcinoma (SCLC) and non-small-cell lung carcinoma (NSCLC) are the two essential lung cancer categories. Among all lung malignancies, NSCLC constitutes 85% and comprises three major types: adenocarcinoma (AC), squamous cell carcinoma (SCC), as well as large cell carcinoma (LCC). Unfortunately, the uppermost NSCLC patients are not diagnosed until late-stage (IIIB-IV) disease is present, and their 5-year survival rate is only 0–10% (Duma et al. 2019).

According to epidemiological studies, smoking is known to be the most common causative factor for developing lung cancer in more than 80% of cases (El-Moselhy and Elrifai 2018). Nevertheless, lung cancer occurs in fewer than 20% of smokers, indicating that genetic factors are the primary contributors that influence lung cancer susceptibility (Eaton et al. 2018). The pathogenic mechanisms of lung cancer are extremely complex and remain poorly understood (Ding et al. 2021). Previous studies demonstrated that the complex combination of chemicals in cigarette smoke causes an inflammatory stress response, producing a continuous source of tumor initiators and promoters in the lung milieu (Landvik et al. 2009). Further research revealed a link between inflammation and tumors, which is largely modulated by various inflammatory cytokines (Tan et al. 2021).

Interleukin-6 (*IL-6*) is a multifactorial interleukin that macrophages and monocytes commonly release (Dutkowska et al. 2021). It is a powerful cytokine that exerts both pro- and anti-inflammatory activities (Campa et al. 2005) and is essential for controlling inflammation, the immune system, and a variety of pathological processes (Kany et al. 2019). Regarding tumorigenesis, *IL-6* conducts as an autocrine growth factor for tumors, which directly prevents apoptosis by deleting cell cycle genes (Silva et al. 2017). Several lines of evidence illustrate that *IL-6* is closely related to a number of cancers, particularly breast, colon, lung, and ovarian cancers (Tan et al. 2021; Almolakab et al. 2022; Kakourou et al. 2015; Chen et al. 2022). Interestingly, *IL-6* has been greatly upregulated in lung cancer, and a molecular link has been suggested via the transcription factor signal transducer and activator of transcription 3 (STAT3) pathway. Furthermore, *IL-6* is considered a reliable prognostic factor for lung cancer patients, as it is correlated with patients' progression, therapy resistance, poor survival, and postoperative complications (Chang et al. 2013).

The *IL-6* gene lies on human chromosome 7 (7p21) in the short arm (Padrón-Morales et al. 2014). Several single-nucleotide polymorphisms (SNPs) were recognized in the promoter of the human *IL-6* gene. Particularly, the − 174G > C (rs1800795) variant is the most commonly studied variant, and it is supposed to functionally regulate the *IL-6* transcription rate and its serum levels (González-Castro et al. 2019). A growing body of research has elucidated that *IL-6* rs1800795 is linked with increased vulnerability to multiple cancers (Harun-Or-Roshid et al. 2021) as well as the prognosis of several malignancies such as NSCLC, ovarian, bladder, neuroblastoma, and breast cancer (Almolakab et al. 2022; Zhai et al. 2017).

Interleukin-*1β* (*IL-1β)* is one of the *IL-1* cytokine families and is predominantly generated by lung epithelia, monocytes, and macrophages (Eaton et al. 2018). It is a cytokine released mainly to promote inflammation and has a master role in cell proliferation, differentiation, and apoptosis control. Interestingly, *IL-1β* leads to the recruitment of numerous inflammatory cytokines, primarily *IL-17A*, *IL-6,* and *IL-22* (Li and Wang 2013). It has been documented that *IL-1β* activates the nuclear factor kappa B (NF-κB) and mitogen-activated protein kinase (MAPK) pathways. However, the certain effect of *IL-1β* as a promotor or progressive factor in tumorigenesis is still not completely understood (Yin et al. 2015). Preclinical studies revealed that mice lacking *IL-1β* have a slower tumor growth and a stronger immune response than wild-type mice (Garon et al. 2020). Further in vivo and in vitro research encouraged the idea that *IL-1β* could induce invasion, metastasis, aggressiveness, immunosuppression, and angiogenesis. Fascinatingly, in lung cancer, *IL-1β* expression has been related to lung cancer development, and its higher levels in serum or tumor tissue have been correlated with a poorer prognosis for lung cancer patients (Zhang and Veeramachaneni 2022).

The *IL-1β* gene is situated in the *IL-1* gene cluster on chromosome 2q (2q14-21). Multiple SNPs of *IL-1β* have been recognized (Li et al. 2015), among them the most significant polymorphisms, -511C/T and -31T/C, which are sited in the promoter section of the gene. These two variants have been in strong linkage disequilibrium and have potential effects on gastric cancer pathogenesis (Eaton et al. 2018). *IL-1β*-511C > T (rs16944) has been proposed to modulate lung cancer risk, and its functional role has been broadly studied (Zienolddiny et al. 2004). Indeed, the identification of polymorphisms in inflammatory genes has attracted much attention from several researchers, particularly for understanding inter-individual variances in lung cancer susceptibility, risk evaluation, as well as prevention or detection (Bhat et al. 2014). On the other hand, the relation between *IL-6* and *IL-1β* SNPs and NSCLC incidence among the Egyptian population has not been reported yet.

Therefore, this research was conducted to determine the impact of *IL-6* rs1800795 and *IL-1β* rs16944 on developing NSCLC in the Egyptian population. Also, the study would examine whether the *IL-6* and *IL-1β* gene SNPs are correlated to NSCLC clinical properties.

## Methods

### Study Populations

Exactly 265 subjects were inducted for this case–control study, divided into 127 primarily diagnosed patients with NSCLC and 138 ethnically matched healthy volunteers as controls. The patient group was enrolled on the basis of being over 18, having a definite histologic or cytological diagnosis of NSCLC (grades I–III), sufficient organ function, visualization by computed tomography (stages I–IV), no prior treatment, accessible clinical records, and no history of cancer or metastatic carcinoma, whereas cases with asthma, bronchitis, pneumonia, lung abscess, tuberculosis, autoimmune disorders, trauma, or other cancers were excluded from the study. The absence of a clinical or family history of cancer or pulmonary diseases was necessary to be considered in the control group.

Using the sample size program (G*Power, Version 3.1.9.6) with a significance level (α) = 0.05, the test power (1-β) = 0.8, and the values of probabilities P1 = 0.06 and P2 = 0.18, the appreciated total samples were 237. These proportion values were obtained from previous studies (Kaanane et al. 2022). Thus, the size of our randomly selected NSCLC samples in this case–control study met the criteria for genotypic statistical analysis.

### Data Collection

When permission from the Medical Ethical Committee was received, all appropriate study participants were given a consent form. All study procedures were completed pursuant to the Helsinki Declaration. Based on patients’ records, we collected their clinical data, including sex, age, smoking status (nonsmoker and smoker), familial history of lung cancer, surgical history, medical history, radiological investigations, histopathologic data (tumor histology and stage), carcinoembryonic antigen (CEA), and the epidermal growth factor receptor (EGFR) gene. Tumor classification was established depending on the American Joint Committee on Cancer (AJCC) staging system guidelines (Detterbeck et al. 2017).

### DNA Extraction and Genotyping

Initially, from each participant, 3 ml of EDTA blood samples was collected for DNA extraction, and then PCR and gel electrophoresis were applied for the detection of *IL-1β* and *IL-6* gene SNPs. The DNA was extracted by the GeneJET DNA Purification Kit (Thermo Fisher Scientific, K 0781, Lithuania). The NanoDropTM 1000 Spectrophotometer was utilized for the assessment of DNA level and purity, and then, it was preserved at − 80 °C until analysis.

The *IL-6* rs1800795 genotyping was evaluated through the amplification refractory mutation system polymerase chain reaction (ARMS-PCR) method**,** where three different primers were used: one common and two other allele-specific primers (Elsaid et al. 2014). The base pair sequencing of primers (Willofort, UK) was forward primer (F): 5′-GAGCTTCTCTTTCGTTCC-3′, reverse primer of the C-allele (R1): 5′-CCTATTGTGTCTTGCC-3′, and reverse primer of the G-allele (R2): 5′-CCCTAGTTGTGTCTTGCG-3′.

The PCR cycling procedures were accomplished via the thermal cycler SimpliAmp™ (Applied Biosystems, USA), from which the denaturation stage at 95 °C for 5 min was accompanied by 30 rounds of denaturation at 94 °C for 30 s, an annealing step at 54 °C for 1 min, extension for 1 min at 72 °C, and finally the extension phase at 72 °C for 7 min. Thereafter, a 2.5% agarose gel electrophoresis was made for the PCR end products and visualized with ethidium bromide. The PCR products of *IL-6* rs1800795 were observed at 230 bp for the G- and C-alleles.

For *IL-1β* rs16944 genotyping**,** the tetra primer amplification refractory mutation system polymerase chain reaction (T-ARMS-PCR) technique was executed (Okayama et al. 2005)**.** The sequence of the primer set (Willofort, UK) was: forward inner primer (C-allele): 5′-CCTGCAATTGACAGAGAGCTACC-3′, reverse inner primer (T-allele): 5′-CTTGGGTGCTGTTCTCTGCCGCA-3′, forward outer primer (Fo): 5′-ATCTGGCATTGATCTGGTTCATCC-3′, and reverse outer primer (Ro): 5′-CTTAACTTTAGGAATCTTCCCACTT-3′.

The thermo-cycling steps started with a denaturation step at 95 °C for 2 min, followed by 30 rounds of denaturation at 95 °C for 30s, annealing at 60 °C for 20s, extension at 72 °C for 30s, and then the final extension stage at 72 °C for 5 min. The electrophoresis of *IL-1β* was then processed on a 2.5% agarose gel and imaged under ultraviolet illumination. The *IL-1β* C-allele was found at 141 bp and the T-allele at 217 bp. When we randomly selected more than 10% of the samples for repeating genotypes, the results were totally consistent.

### Statistics

Statistical tests were computed by The IBM Statistical Package for Social Science (SPSS; version 25.0). The quantitative variables were presented as mean ± standard error (M ± SE) and the qualitative variables as numbers and percentages (N %). The qualitative data were compared using Fisher’s exact test, while the quantitative data were compared by the Mann–Whitney U-test. For testing for Hardy–Weinberg equilibrium (HWE), the observed and expected genotypic counts of both *IL-6* rs1800795 and *IL-1β* rs16944 were compared among the case and control groups by the Chi-square test. The allelic and genotypic frequencies of *IL-6* and *IL-1β* SNPs were figured by Fisher’s exact test.

Logistic regression analysis was utilized to assess the link between *IL-6* and *IL-1β* studied variants and NSCLC risk in different genetic models (overdominant, dominant, recessive, codominant, and allelic) (Elsaid et al. 2019), with adjustment for age, sex, smoking, and family history. Thus, odds ratios (ORs) and 95% confidence intervals (CIs) were elucidated by logistic regression. In all statistical tests, a two-sided *P* value less than 0.05 indicated a significant association. Logistic regression analysis was also used to stratify clinical data, including age, gender, smoking, and pathological types. Statistical graphics were performed using Origin Lab software (version 2022).

## Results

### Principal Characteristics of All Subjects

The overall number of subjects in this study was 265, consisting of 127 NSCLC patients and 138 healthy volunteers. The average age of NSCLC cases was 55.8 (± 1.03) years, with males accounting for 59.8% and females comprising 40.2%. The other control group had a matched average age of 53.4 (± 0.88) years and percentages of 66.7% for males and 33.3% for females. NSCLC cases included 65 (51.2%) smokers, but controls included about 33 (23.9%) smokers, resulting in a highly significant difference (*p* < 0.001). Also, family history resembled 14 (11%) in NSCLC cases while it was absent (0%) in healthy subjects, thus a remarkable statistical significance appeared (*p* < 0.001). According to histopathological characteristics, the majority of cases (78%) were classified as AC and presented in late stages III and IV (96%). Detailed clinical data for NSCLC cases and controls are shown in Table 1.

**Table 1:** Characteristics of the study subjects

Parameter		Cases (n = 127) n (%)	Controls (n = 138) n (%)	*P* value
Age (years)	Mean ± SE	55.8 ± 1.03	53.4 ± 0.88	0.091^a^
Sex	Female/Male	51 (40.2)/76 (59.8)	46 (33.3)/92 (66.7)	0.25^b^
Smoking	Smokers/Nonsmokers	65 (51.2)/62 (48.8)	33 (23.9)/105 (76.1)	** < 0.001**^b^
Symptoms				
Cough	Positive/Negative	49 (38.6)/78(61.4)		
Dyspnea	Positive/Negative	44 (34.6)/83(65.4)		
Chest pain	Positive/Negative	40 (31.5)/87(68.5)		
Hemoptysis	Positive/Negative	9 (7.1)/118(92.9)		
Family history	Positive/Negative	14 (11)/113(89)	0 (0)/138 (100)	** < 0.001**^b^
Surgical history	Positive/Negative	45 (35.4)/82(64.5)	36 (26.1)/102 (73.9)	0.099^b^
Medical history	Positive/Negative	66 (52)/61(48)	57 (41.3)/81 (58.7)	0.082^b^
Tumor size (T)	T1, T2	12 (9.5)		
	T3, T4	115 (90.6)		
Lymph node (N)	N0, N1	31 (24.4)		
	N2, N3	96 (75.6)		
Stage	Stage 1, 2	5 (4)		
	Stage 3, 4	122 (96)		
Grade	Mild	3 (2.4)		
	Moderate, High	124 (97.6)		
Histological types	Adenocarcinoma	99 (78)		
	Squamous cell carcinoma	13 (10.2)		
	Large cell carcinoma	15 (11.8)		
CEA (ng/mL)	Mean ± SE	35.47 ± 6.26		
	Positive/Negative	80 (63)/47(37)		
EGFR status	Wild/Mutant	96 (75.6)/31 (24.2)		

^a^Mann–Whitney U-test

Bold values signify *p* < 0.05

^b^Fisher’s exact test

*SE* standard error, *CEA* carcinoembryonic antigen, *EGFR* epidermal growth factor receptor

### Relationship Between the IL-6 (rs1800795) Variant and NSCLC Vulnerability

**Fig. 1:**
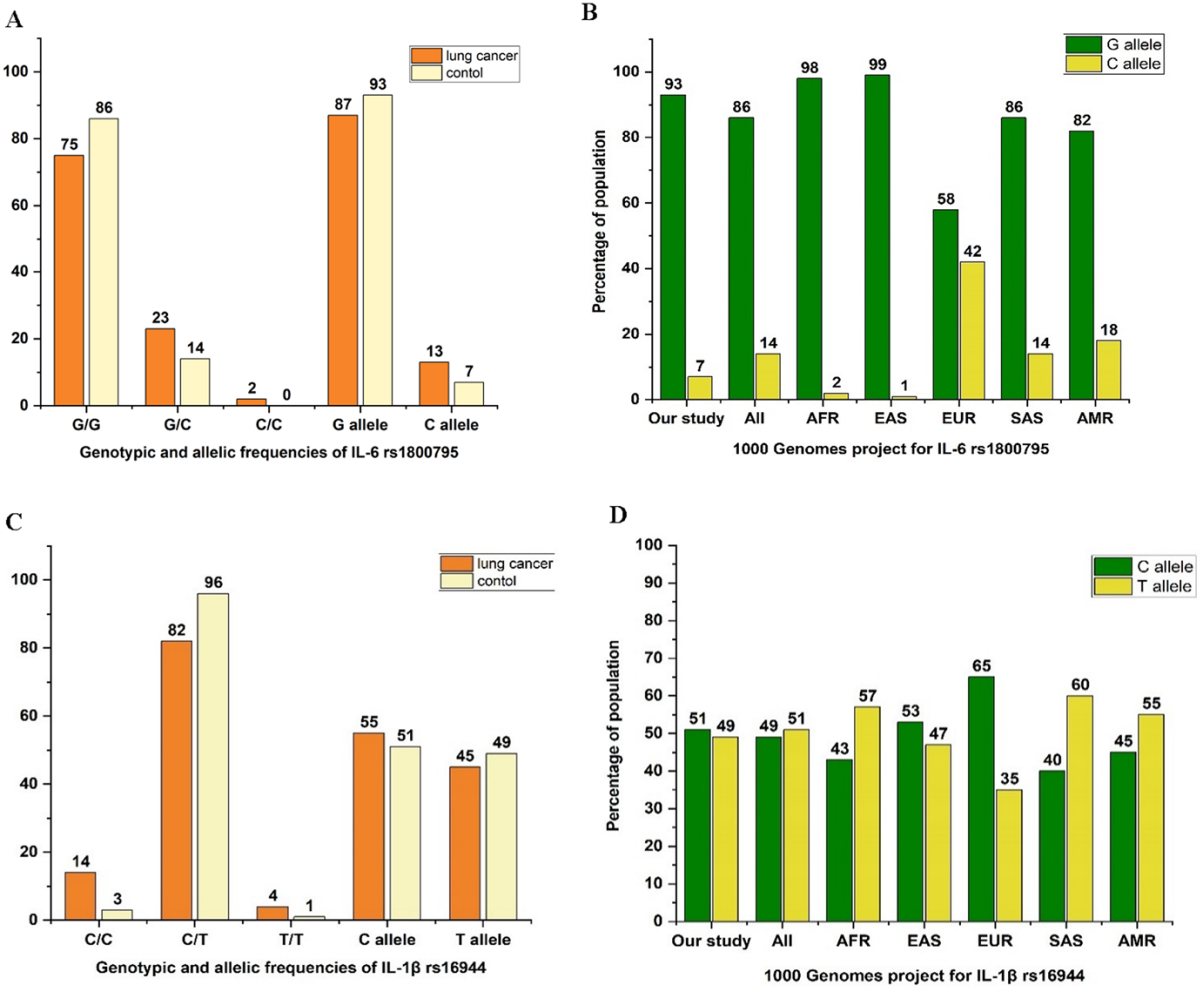
Allelic and genotypic frequencies of the study population. **A** Genotype and allele frequencies of the *IL-6* (rs1800795) variant among NSCLC patients and cancer-free controls. **B** Allelic frequencies of the *IL-6* (rs1800795) variant in the current study compared to different populations based on the 1000 Genome Project Phase 3 (https://www.internationalgenome.org/). **C** Genotype and allele frequencies of the *IL-1β* (rs16944) variant among NSCLC patients and cancer-free controls. **D** Allelic frequencies of the *IL-1β* (rs16944) variant in the current study compared to different populations based on the 1000 Genome Project Phase 3. *AFR* Africa, *AMR* America, *EAS* East Asia, *EUR* Europe, *SAS* South Asia

The expected and observed frequencies of *IL-6*-174G > C followed the Hardy–Weinberg equation among NSCLC patients and healthy volunteers (*p* > 0.05). The effect of the *IL-6* rs1800795 variant on the occurrence of NSCLC was evaluated using various inheritance models with adjustments for age and gender, as presented in Table 2. The results indicated a low frequency of the rare genotype ‘CC’ (1.6%) among NSCLC patients and its absence among controls (0%), with a non-significant variance in the recessive model (*p* = 0.999). In addition, the minor allele frequency (MAF) of *IL-6*-174G > C in the healthy individuals was 0.07 (Fig. 1A), which was correlated with other ethnic populations, especially African (0.02) and South Asian (0.14) (Fig. 1B).

**Table 2:** Distribution of *IL-6* (rs1800795) and *IL-1β* (rs16944) polymorphisms among Egyptian patients with NSCLC versus controls

Gene	Polymorphism genotype	Cases (n = 127)	Controls (n = 138)	Model	Genotype	OR (95% CI)	*P* value^a^	Adjusted OR (95% CI)	*P* value^b^
IL-6	**GG**	95 (74.8)	119 (86.2)						
	**GC**	30 (23.6)	19 (13.8)	Codominant	GC vs. GG	**1.97 (1.04**–**3.73)**	**0.035**	**2.2 (1.08**–**4.37)**	**0.028**
	**CC**	2 (1.6)	0 (0)		CC vs. GG	NA (0.00–NA)	0.999	NA (0.00–NA)	0.999
	**CC + GC**	32 (25.2)	19 (13.8)	Dominant	CC + GC vs. GG	**2.11 (1.12–3.95)**	**0.02**	**2.34 (1.17**–**4.6)**	**0.015**
	**GG + GC**	125 (98.4)	138 (100)	Recessive	CC vs. GG + GC	NA (0.00–NA)	0.999	NA (0.00–NA)	0.999
	**GG + CC**	97 (76.4)	119 (86.2)	Overdominant	GC vs. GG + CC	**1.93 (1.02**–**3.65)**	**0.041**	**2.1 (1.06–4.27)**	**0.033**
	**G**	220 (86.6)	257 (93.1)	Allelic	C vs. G	**2.09 (1.15**–**3.76)**	**0.014**	**2.28 (1.2**–**4.33)**	**0.011**
	**C**	34 (13.4)	19 (6.9)						
	**HWE**^**c**^	X^2^ = 0.04, *P* = 0.978	X^2^ = 0.75, *P* = 0.685						
IL-1β	**CC**	18 (14.2)	4 (2.9)						
	**CT**	104 (81.9)	132 (95.7)	Codominant	CT vs. CC	**0.17 (0.06**–**0.53)**	**0.002**	**0.16 (0.05**–**0.56)**	**0.004**
	**TT**	5 (3.9)	2 (1.4)		TT vs. CC	0.75 (0.27–1.99)	0.558	1.15 (0.12–11.2)	0.901
	**TT + CT**	109 (85.8)	134 (97.1)	Dominant	TT + CT vs. CC	**0.18 (0.05**–**0.55)**	**0.003**	**0.17 (0.05**–**0.59)**	**0.005**
	**CT + CC**	122 (96.1)	136 (98.6)	Recessive	TT vs. CT + CC	2.78 (0.53–14.6)	0.226	6.14 (0.96–39.1)	0.055
	**TT + CC**	23 (18.1)	6 (4.3)	Overdominant	CT vs. TT + CC	**0.21 (0.08**–**0.52)**	**0.001**	**0.15 (0.05**–**0.44)**	**0.001**
	**C**	140 (55.1)	140 (50.7)	Allelic	T vs. C	0.84 (0.59–1.18)	0.312	0.88 (0.61–1.28)	0.516
	**T**	114 (44.9)	136 (49.3)						
	**HWE**^**c**^	X^2^ = 54.5, *P* < **0.001**	X^2^ = 115.1, *P* < **0.001**						

Bold values express the *p* < 0.05

^a^*p* value for the genetic inheritance models was obtained by logistic regression

^b^*p* value with adjusted age, sex, smoking, and family history

^c^HWE was performed by Chi-square test

*OR* odds ratio, *CI* confidence interval 95%, *HWE* Hardy–Weinberg equilibrium; *NA* not applicable

Remarkably, *IL-6* ‘CC + GC’ genotypes amplified the NSCLC risk in the dominant model (adjusted, OR = 2.34, *p* = 0.015), and *IL-6* ‘GC’ genotype raised the NSCLC risk in the overdominant model (adjusted, OR 2.1, *p* = 0.033). Under the allele model, the mutant allele ‘C’ of rs1800795 significantly increased NSCLC risk by 2.28-fold (adjusted, OR 2.28, *p* = 0.011).

### Relationship Between the IL-1β (rs16944) Variant and NSCLC Vulnerability

When *IL-1β*-511C > T (rs16944) frequencies were checked for genetic equilibrium using the Hardy–Weinberg law, they significantly deviated from expected frequencies (*p* < 0.001) in both cases and controls. This significant deviation may be attributed to the higher frequency of the protective heterozygote that may be related to the Egyptian population. This observation would require further confirmatory studies with a larger sample of controls.

Moreover, the minor allele frequency (MAF) of *IL-1β*-511C > T in the healthy individuals was 0.49 (Fig. 1C), which was related to other ethnic populations, mainly East Asian (0.47) and American (0.55) (Fig. 1D). Table 2 shows the resultant statistics using different models of inheritance of the *IL-1β* (rs16944) SNP among NSCLC patients and controls. The results of rs16944 revealed that specifically patients with heterozygous genotype ‘CT’ were less prone to NSCLC incidence than those with homozygous genotypes ‘TT + CC’ in the overdominant model (adjusted, OR 0.15, *p* = 0.001).

### Stratification Analysis of IL-6 (rs1800795) Variant and NSCLC Risk

According to Table 3**,** stratifying tests by age, gender, smoking, and histological subtypes were performed via the three selected models: dominant, codominant heterozygous, and overdominant; the other models have not been included because of the rare *IL-6* CC genotype numbers. Regarding sex, females carrying the *IL-6*-C-allele were more likely to have NSCLC susceptibility compared to males in the dominant model (adjusted, OR 7.0, *p* = 0.005, CC + GC vs. GG). Additionally, analysis of the histological subtypes of NSCLC identified that *IL-6-*C-allele carriers apparently elevated the odds of AC under the dominant model (adjusted the, OR 2.97, *p* = 0.003, CC + GC vs. GG).

**Table 3:** Association between *IL-6* rs1800795 and NSCLC using stratification analyses

Variable	Cases/Controls			Adjusted OR (95% CI); *P* value^a^		
	GG	GC	CC	GC vs. GG	CC + GC vs. GG	GC vs. GG + CC
Sex						
Female	36/43	14/3	1/0	**6.19 (1.57**–**24.4); 0.009**	**7.0 (1.79**–**27.4); 0.005**	**5.9 (1.51**–**23.1); 0.011**
Male	59/76	16/16	1/0	1.29 (0.54–3.11); 0.569	1.35 (0.56–3.2); 0.501	1.27 (0.53–3.06); 0.594
Age						
< 55	47/67	10/7	1/0	0.44 (0.15–1.31); 0.143	0.39 (0.14–1.16); 0.09	0.45 (0.15–1.35); 0.157
> = 55	48/52	20/12	1/0	0.46 (0.18–1.16); 0.099	0.44 (0.17–1.11); 0.083	0.46 (0.18–1.18); 0.107
Smoking						
Smoker	47/30	17/3	1/0	0.34 (0.09– 1.34); 0.123	0.33 (0.09– 1.26); 0.105	0.35 (0.09– 1.37); 0.132
Nonsmoker	48/89	13/16	1/0	0.54 (0.23– 1.26); 0.154	0.50 (0.22– 1.16); 0.108	0.55 (0.23– 1.29); 0.166
Histological types						
Adenocarcinoma	69/120	29/19	1/0	**2.89 (1.41**–**5.93); 0.004**	**2.97 (1.45**–**6.07); 0.003**	**2.85 (1.39**–**5.85); 0.004**
Squamous cell carcinoma	12/119	0/19	1/0	NA (0.00–NA); 0.998	1.04 (0.11–9.86); 0.971	NA (0.00– NA); 0.998
Large cell carcinoma	14/119	1/19	0/0	0.52 (0.06–4.55); 0.558	0.52 (0.06–4.55); 0.558	0.52 (0.06–4.55); 0.558

Bold values express the *p* < 0.05

^a^*p* value was obtained by logistic regression after adjustment for age, sex, smoking, and family history

*OR* odds ratio, *CI* confidence interval 95%; *NA* not applicable;

### Stratification Analysis of IL-1β (rs16944) Variant and NSCLC Risk

As shown in Table 4, the differential investigations revealed that patients with heterozygous genotype ‘CT’ were significantly correlated to a lower NSCLC incidence for males in the overdominant model (adjusted, OR = 0.17, *p* = 0.006). Also, strata of age indicated that patients under the age of 55 were less likely to develop NSCLC with the overdominant model (for CT vs. TT + CC, adjusted, OR = **0.1**7, *p* = **0.00**4). The *IL-1β*-511 CT genotype had a significant protective effect on the NSCLC incidence for nonsmokers, notably in the overdominant model (for CT vs. TT + CC, adjusted, OR = **0.1**0, *p* = **0.00**1). However, the *IL-1β*-511 CT genotype was linked to a decreased susceptibility to all lung cancer subtypes (*p* < 0.01). Thus, the protective role of the *IL-1β*-511 CT genotype on NSCLC occurrence has been similar across histological subtypes.

**Table 4:** Association between *IL-1β* rs16944 and NSCLC using stratification analyses

Variable	Cases/Controls			Adjusted OR (95% CI); *P* value^a^				
	CC	CT	TT	TT vs. CC	CT vs. CC	TT + CT vs. CC	TT vs. CT + CC	CT vs. CC + TT
Sex								
Female	8/1	43/45	0/0	NA (0.00–NA); 0.999	0.13 (0.01–1.16); 0.068	0.13 (0.01–1.16); 0.068	NA (0.00–NA); 0.999	0.1 (0.02–1.16); 0.06
Male	10/3	61/87	5/2	1.24 (0.13–11.8); 0.85	**0.20 (0.04–0.95); 0.044**	0.23 (0.05–1.07); 0.061	6.0 (0.93–38.6); 0.059	**0.17 (0.04**–**0.61); 0.006**
Age								
< 55	12/3	43/69	3/2	0.61 (0.05–7.58); 0.698	**0.16 (0.04**–**0.65); 0.01**	**0.17 (0.04**–**0.69); 0.013**	3.63 (0.53–24.8); 0.189	**0.17 (0.05**–**0.57); 0.004**
> = 55	6/1	61/63	2/0	NA (0.00–NA); 0.999	0.29 (0.03–2.87); 0.295	0.30 (0.03–2.94); 0.30	NA (0.00–NA); 0.999	0.23 (0.02–2.01); 0.183
Smoking								
Smoker	8/2	54/31	3/0	NA (0.00–NA); 0.999	0.46 (0.08–2.49); 0.37	0.49 (0.09–2.61); 0.40	NA (0.00–NA); 0.999	0.34 (0.07–1.7); 0.188
Nonsmoker	10/2	50/101	2/2	0.61 (0.02–15.7); 0.765	**0.086 (0.01–0.47); 0.005**	**0.09 (0.02–0.51); 0.006**	NA (0.00–NA); 0.999	**0.10 (0.03–0.41); 0.001**
Histological types								
Adenocarcinoma	12/4	82/133	5/2	3.5 (0.23–55.1); 0.368	**0.26 (0.07**–**0.94); 0.04**	**0.27 (0.07**–**1.0); 0.05**	**9.3 (1.4**–**60.6); 0.02**	**0.18 (0.06**–**0.56); 0.003**
Squamous cell carcinoma	3/4	10/132	0/2	NA (0.00–NA); 0.999	**0.07 (0.007**–**0.77); 0.029**	**0.07 (0.007**–**0.76); 0.029**	NA (0.00–NA); 0.999	**0.09 (0.008**–**0.84); 0.036**
Large cell carcinoma	3/4	12/132	0/2	NA (0.00–NA); 0.999	**0.13 (0.02**–**0.85); 0.033**	**0.14 (0.02**–**0.85); 0.033**	NA (0.00–NA); 0.999	0.18 (0.03–1.02); 0.053

Bold values reflect a *p* < 0.05

^a^*p* value was analyzed by logistic regression after adjustment for age, sex, smoking, and family history

*OR* odds ratio, *CI* confidence interval 95%; *NA* not applicable

### Association of IL-6 and IL-1β Variants and the NSCLC Clinical Features

Tables 5 and 6 display possible leverage of the *IL-6* rs1800795 and *IL-1β* rs16944 variants on the clinicopathological characteristics of NSCLC patients**.** Remarkably, Patients who presented with dyspnea and chest pain were highly correlated with the *IL-6* SNP under the dominant model (dyspnea,* p* = 0.035; chest pain,* p* = 0.025) and the allelic model (dyspnea,* p* = 0.043; chest pain,* p* = 0.024), respectively. It was obvious that the *IL-6* SNP had a high connotation with tumor sizes in NSCLC patients over the allelic (*p* = 0.05) and dominant models (*p* = 0.036). Besides, histological types of NSCLC were greatly correlated with the *IL-6* variant under the dominant model (*p* = 0.044), while the *IL-1β* rs16944 frequencies among NSCLC patients showed a non-significant correlation with the clinical data of NSCLC (all *p* > 0.05).

**Table 5:** *IL-6* (rs1800795) variant and clinical parameters of NSCLC patients

Parameter		CC + GC (n = 32)	GG (n = 95)	*P* value^a^	C-allele (n = 34)	G-allele (n = 220)	*P* value^a^
Age	Age < 55/Age > = 55	11/ 21	47/48	0.138	12/22	104/116	0.192
Sex	Female/Male	15/17	36/59	0.37	16/18	86/134	0.378
Smoking	Smokers/Non smokers	18/14	47/48	0.507	19/15	111/109	0.556
Symptoms							
Cough	Positive/Negative	13/19	36/59	0.784	13/21	85/135	0.964
Dyspnea	Positive/Negative	16/16	28/67	**0.035**	17/17	71/149	**0.043**
Chest pain	Positive/Negative	5/27	35/60	**0.025**	5/29	75/145	**0.024**
Hemoptysis	Positive/Negative	2/30	7/88	1.0	2/32	16/204	1.0
Family history	Positive/Negative	2/30	12/83	0.363	2/32	26/194	0.391
Surgical history	Positive/Negative	10/22	35/60	0.567	12/22	78/142	0.985
Medical history	Positive/Negative	18/14	48/47	0.575	19/15	113/107	0.624
T	T1, T2	0	12	**0.036**	0	24	**0.05**
	T3, T4	32	83		34	196	
N	N0, N1	8	23	0.928	8	54	0.898
	N2, N3	24	72		26	166	
Stage	Stage 1, 2	0	5	0.329	0	10	0.367
	Stage 3, 4	32	90		34	210	
Grade	Mild	1	2	1.0	2	4	0.185
	Moderate, High	31	93		32	216	
Histological types	Adenocarcinoma	30	69	**0.044**	31	167	0.123
	Squamous cell carcinoma	1	12		2	24	
	Large cell carcinoma	1	14		1	29	
CEA (ng/mL)	Positive/Negative	21/11	59/36	0.721	22/12	138/82	0.824
EGFR status	Wild/Mutant	26/6	70/25	0.389	28/6	164/56	0.324

Bold values signify *p* < 0.05

^a^Fisher’s exact test

*CEA* carcinoembryonic antigen, *EGFR* epidermal growth factor receptor

**Table 6:** *IL-1β* (rs16944) variant and clinical parameters of NSCLC patients

Parameter		TT + CT (n = 109)	CC (n = 18)	*P* value^a^	T-allele (n = 114)	C-allele (n = 140)	*P* value^a^
Age	Age < 55/Age > = 55	46/63	12/6	0.054	49/65	67/73	0.438
Sex	Female/Male	43/66	8/10	0.689	43/71	59/81	0.474
Smoking	Smokers/Non smokers	57/52	8/10	0.537	60/54	70/70	0.676
Symptoms							
Cough	Positive/Negative	39/70	10/8	0.11	40/74	58/82	0.302
Dyspnea	Positive/Negative	39/70	5/13	0.509	41/73	47/93	0.69
Chest pain	Positive/Negative	35/74	5/13	0.714	37/77	43/97	0.76
Hemoptysis	Positive/Negative	8/101	1/17	1.0	8/106	10/130	0.96
Family history	Positive/Negative	10/99	4/14	0.113	10/104	18/122	0.301
Surgical history	Positive/Negative	40/69	5/13	0.464	42/72	48/92	0.672
Medical history	Positive/Negative	57/52	9/9	0.857	60/54	72/68	0.849
T	T1, T2	10	2	1.0	11	13	0.922
	T3, T4	99	16		103	127	
N	N0, N1	25	6	0.341	27	35	0.808
	N2, N3	84	12		87	105	
Stage	Stage 1, 2	3	2	0.147	3	7	0.519
	Stage 3, 4	106	16		111	133	
Grade	Mild	2	1	0.37	2	4	0.694
	Moderate, High	107	17		112	136	
Histological types	Adenocarcinoma	87	12	0.426	92	106	0.63
	Squamous cell carcinoma	10	3		10	16	
	Large cell carcinoma	12	3		12	18	
CEA (ng/mL)	Positive/Negative	69/40	11/7	0.858	72/42	88/52	0.961
EGFR status	Wild/Mutant	82/27	14/4	1.0	86/28	106/34	0.959

^a^Fisher’s exact test

*CEA* carcinoembryonic antigen, *EGFR* epidermal growth factor receptor

### Expression of IL-6 and IL-1β Genes Plus Their SNPs

**Fig. 2:**
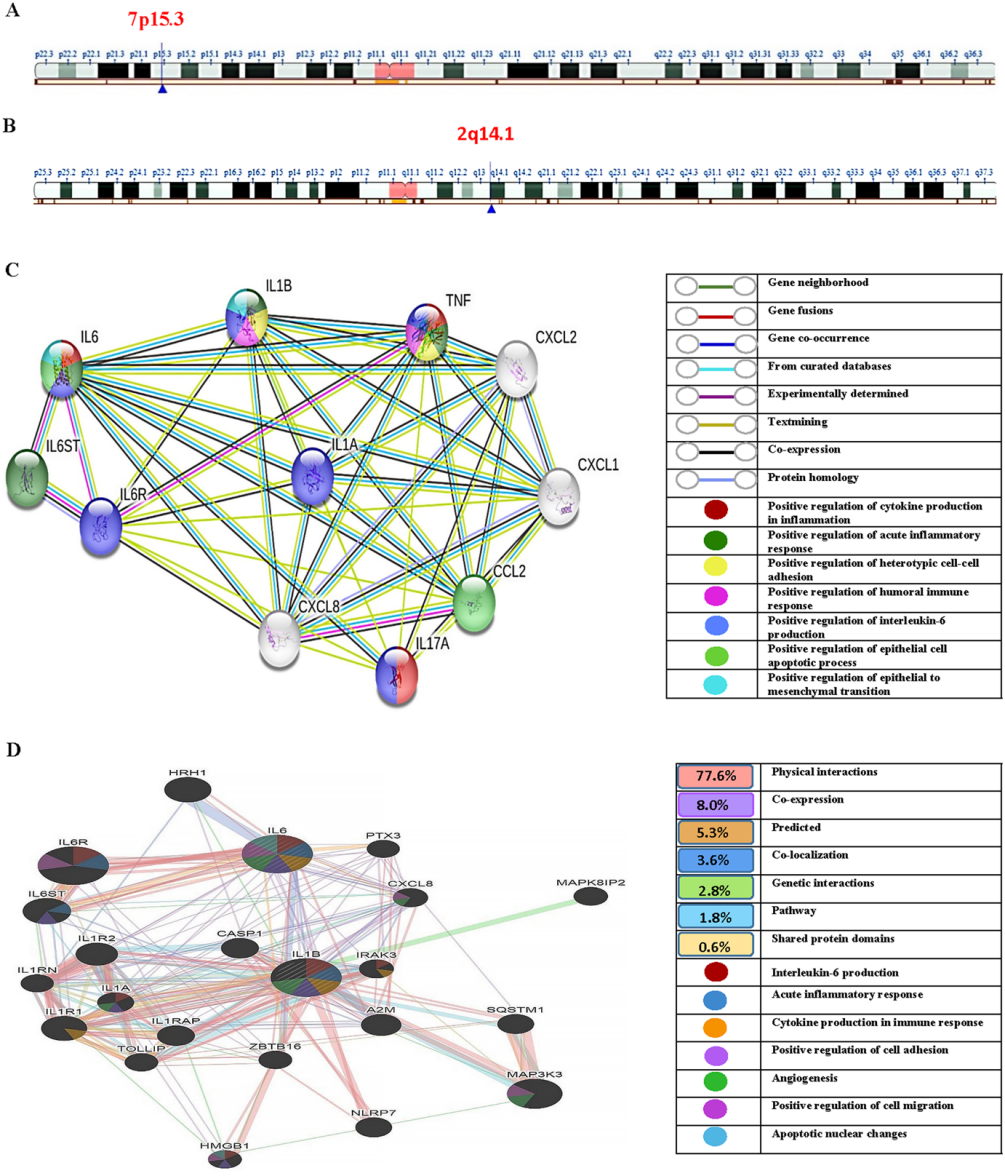
In silico investigations of *IL-1β* and *IL-6*. **A** The *IL-6* gene is located at chromosome 7p15.3 and spans 6,119 bases (chr7: 22,725,884–22,732,002) along the plus strand. The *IL-6* (rs1800795) variant is located on chromosome 7: 22,727,026. **B** The *IL-1β* gene is located at chromosome 2q14.1 and spans 7,066 bases (chr2: 112,829,751–112,836,816) along the minus strand. The *IL-1β* (rs16944) variant is located at chromosome 2: 112,837,290 (NCBI database; human genome assembly GRCh38.p14) **C** The STRING database result reveals 11 nodes (proteins) interconnected by 46 edges (interactions) with a *p-value* equal to 9.88e–8 and an average node degree of 8.36. *TNF*: Tumor necrosis factor; *IL1B* Interleukin-1 beta; *IL1A* Interleukin-1 alpha; *CXCL* Chemokine (C-X-C motif) ligand; *CCL2* Chemokine (C–C motif) ligand 2; *IL17A* Interleukin 17A; *IL6* Interleukin-6; *IL6R* Interleukin-6 receptor; *IL6ST* Interleukin-6 signal transducer. **D** The GENEMANIA database result shows 22 gene interactions responsible for different actions, mainly acute inflammation, cytokine production, and regulation of tumorigenesis

The genetic structure of *IL-6* as presented in **(**Fig. 2A**)** is mapped at chromosome 7p15.3 and assigned 6,119 bases (chr7: 22,725,884–22,732,002) within the forward strand. The *IL-6* (rs1800795) variant is located on chromosome 7: 22,727,026. Otherwise, the *IL-1β* gene is located on chromosome 2q14.1 and covers 7066 bases (chr2: 112,829,751–112,836,816) along the minus strand. The *IL-1β* (rs16944) variant is located at chromosome 2: 112,837,290 (NCBI database; human genome assembly GRCh38.p14) **(**Fig. 2B**).**

The GTEx database (https://gtexportal.org/home/) predicted that normal lung tissues have a differential expression of the *IL-6* rs1800795 variant (*p* = 7.1 × 10^–39^) **(**Fig. S1**).** Using the GEPIA database (http://gepia.cancer-pku.cn/), the *IL-6* gene expression was found to be considerably lower in both AC and SCC tissues comparable with para-tumor tissues (*p* < 0.01) **(**Fig. S2A and S2B). In contrast, a non-significant difference was observed in *IL-1β* expression in both AC and SCC tissues compared to para-tumor tissues (all *p* > 0.01) (Fig. S3A and S3B). The prognosis of NSCLC was non-significantly correlated with either the *IL-6* or *IL-1β* genes (*p* > 0.05) (Figs. S2C, S2D, S3C, and S3D). Interestingly, there was a noteworthy correlation between both genes in AC (*p* < 0.001**, **Fig. S4A) and SCC (*p* = 1.0 × 10^–5^, Fig. S4B). Meanwhile, in silico investigation for *IL-1β* and *IL-6* was accomplished by the STRING database version 11.5 (https://string-db.org/). *IL-6* protein shares positive regulation of cytokine production, acute inflammatory response, apoptotic process, and epithelial to mesenchymal transition, whereas the *IL-1β* protein shares positive regulation of *IL-6* production, humoral immune response, and cell–cell adhesion **(**Fig. 2C**).** Gene–gene functions using the GENEMANIA database (https://genemania.org/) were also computed for *IL-6* and *IL-1β*, which identified physical, co-expression, predicted, and co-localization interactions between the two genes (Fig. 2D).

## Discussion

In light of the growing research that focuses on the etiologic role of inflammation in lung malignancies, this study was directed to manifest the impact of inflammatory genes, mainly genetic variants of *IL-6* (rs1800795) and *IL-1β* (rs16944), on NSCLC occurrence in the Egyptian subjects.

Several reports have focused on exploring the pro-tumorigenic functions of IL-6 (Yang 2017; Huang et al. 2022). IL-6 can activate several signaling pathways, including mTOR, STAT3, Janus tyrosine kinase (JAK)/STAT, and NF-κB (Brábek et al. 2020). Its activation of JAK/STAT3 is the predominant pathway driven by multiple cancer types (Ke et al. 2020; Masjedi et al. 2018; Lin et al. 2020). Upon binding of IL-6 to its IL-6 receptor (IL-6R) and the signal transducer subunit glycoprotein 130 (gp130), JAK2 tyrosine kinases are activated, leading to STAT3 recruitment, phosphorylation, and dimerization. The STAT3 transcription factors are then translocated to the nucleus, affecting the transcription of different genes that contribute to multifaceted activities, including inflammation (IL-6, IL-17, IL-23, Cox2), antiapoptosis (Bcl-2, survivin, Mcl-1), proliferation (c-Myc, Cyclin D1, Cyclin B), neovascularization (VEGF), migration (MMP2, MMP9), and cell adhesion (ICAM-1, TWIST1) (Browning et al. 2018; Chonov et al. 2019; Johnson et al. 2018).

This study identified a significant connection between the *IL-6* (rs1800795) variant and NSCLC vulnerability. Furthermore, the *IL-6* (rs1800795) SNP increased the risk of NSCLC in females. Our results were consistent with Kaanane et al. (2022), who discovered that the *IL-6*-174G > C (CC) genotype could boost lung cancer possibility in their Moroccan carriers. A meta-analysis study has confirmed that *IL-6*-174G > C is an independent risk factor for lung cancer prevalence in Caucasian (OR 1.04, *p* < 0.001) and Asian populations (OR 1.05, *P* = 0.003) (Peng et al. 2018).

On the contrary, negative studies have been reported by Gao et al. (2020)**,** which revealed no genotypic distribution differences for *IL-6* rs1800795 in lung cancer patients using meta-analysis. Eaton et al. (2018) designed a case–control study, which revealed a non-significant interrelation between the genotypes of *IL-6* rs1800795 and susceptibility to lung cancer. Also, Liu et al. (2015) established a meta-analysis study that showed that genotypic and allelic frequencies of the *IL-6* rs1800795 variant were not substantially related to lung cancer susceptibility. A comprehensive meta-analysis showed that rs1800795 was generally linked to the cancer risk of both Africans and Asians via different inherited models. However, the stratifying analysis based on cancer subtypes revealed no significant relationship with lung cancer, which may be due to heterogeneity (Harun-Or-Roshid et al. 2021)**.** This was observed in Asians, who have lower or even absent *IL-6*-174C-allele frequencies than Caucasians (Tian et al. 2015).

Several genetic association studies revealed that the *IL-6*-174G > C SNP is functionally important in controlling the transcription of the *IL-6* gene, but the exact mechanism is still unclear. It was reported that the − 174C-allele is related to increased expression levels of the *IL-6* gene when compared to the -174G-allele (González-Castro et al. 2019; Huang et al. 2022). Therefore, the *IL-6*-174G > C SNP could affect the NSCLC risk by upregulating the transcription of IL-6 levels. However, *IL-6* transcription is a multifactorial process that depends on more than one SNP regulatory site, so it must be taken into consideration (Tumu et al. 2013).

This study demonstrated that dyspnea and chest pain in NSCLC patients are greatly associated with the *IL-6*-174G > C SNP. In a previous study, Wang et al. ( 2017) demonstrated that *IL-6* rs1800795 was associated with fatigue throughout and after the treatment of cancer. However, our work has primarily revealed significant associations between the *IL-6* SNP and patient symptoms of NSCLC.

Surprisingly, our findings recognized that the *IL-6* variant is meaningfully affected on the tumor size in NSCLC patients. In the same way, Koh et al. (2012) illustrated the prognostic effect of *IL-6* on lung cancer patients both in serum and tissues and found that its expression in tissues was positively correlated with pathological data for T status, N status, and staging. Thus, this observation referred to the possibility that *IL-6* variant could be a valuable marker for NSCLC prognosis.

Additionally, the present study proposes that *IL-6* rs1800795 specifically increases the risk of AC. Balabko et al. (2014) observed that AC exhibits greater IL-6R expression in comparison with SCC, which is related to the interconnection between Th17 lymphocytes, IL-6R, and the pSTAT3/BATF/RorγT-axis. In contrast, Ding et al. (2021) conducted a meta-analysis that revealed non-significant correlations between the rs1800795 SNP and lung cancer clinical manifestations, comprising histology subtypes, sex, and smoking. Thus, the observed differences in *IL-6* rs1800795 distribution among NSCLC subtypes may support the specific impact of *IL-6* among lung cancer subtypes.

On the other hand, IL-1β production occurs mainly in macrophages as well as monocytes, adipocytes, dendritic cells, fibroblasts, and B cells. It is initiated by lipopolysaccharides (LPS) through toll-like receptors (TLRs), TNFα through the TNF receptors, or IL-1β itself. It is produced as an inactive pro-IL-1β, which is proteolyzed into an active IL-1β cytokine and then prepared for secretion (Habanjar et al. 2023). Once released, IL-1β interacts with its IL-1 receptor, driving the NF-κB and MAPK signaling cascades to activate various target genes. The granulocyte–macrophage colony-stimulating factor is one of the downstream effects released via IL-1β, which enhances the synthesis of M2-type macrophages and myeloid-derived suppressor cells (MDSCs) for promoting tumor invasion, immune escape, and tumor growth (Garon et al. 2020).

The current study observed a protective effect for the *IL-1β*-511(CT) heterozygous genotype on NSCLC Egyptian subjects. In accordance with our consequences, Zienolddiny et al. (2004) confirmed that the *IL-1β* polymorphisms were markedly linked to lung cancer susceptibility in the Norwegian population, where the wild-type genotype *IL-1β*-511CC raised the odds of lung cancer (OR 2.5) compared with the less frequent genotype *IL-1β-*511TT. In the case–control study, Eaton et al. (2018) discovered that both the CT and TT genotypes of rs16944 decreased the lung cancer risk (OR 0.74, OR 0.71, *p* = 0.03), respectively, compared to the wild-type genotype CC. A recent meta-analysis revealed that the rs16944 (T) allele significantly decreased lung cancer susceptibility (*p* = 0.04), and the homozygote genotype (TT) also decreased lung cancer exposure under the recessive model (*p* = 0.04) (Ding et al. 2021)**.** On the contrary, further studies showed that there was a non-significant effect of rs16944 on the evolution of lung cancer under different models (Li and Wang 2013; Pérez-Ramírez et al. 2017; Kiyohara et al. 2014).

It has been found that the *IL-1β* SNP (rs16944) largely affects the transcription of *IL-1β* among various diseases, where the major allele (C) is related to higher expression levels compared to the minor allele (T) (Kaarvatn et al. 2012). This can in part interpret our findings that the *IL-1β* SNP (rs16944) may be a protective factor by controlling the levels of IL-1β in lung cancer patients. However, many more studies would be mandatory to prove this concept.

According to our findings, patients under the age of 55, males, and nonsmokers with the *IL-1β*-511(CT) heterozygote genotype have lowered the risk of NSCLC via different models. A previous study performed on Chinese populations over the age of 63 showed that the rs16944T > C (CC) homozygous variant significantly raised the risk of NSCLC (adjusted, OR 1.482, *P* = 0.014) (Li et al. 2015). Another case–control study noted that subgroup analysis of rs16944, rs1143634, and rs1143623 *IL-1β* SNPs greatly affected lung cancer risk, particularly for males, current smokers, and SCC subdivisions (Eaton et al. 2018).

Furthermore, in our study, the *IL-6* (rs1800795) variant is expected to be differentially expressed in normal lung tissues using a database. Our findings also suggest that levels of the *IL-6* gene are significantly lower in both AC and SCC tumor tissues than in para-tumor tissues. Recently, Dutkowska et al. (2021) revealed a downregulation of *IL-6* mRNA levels in lung tumor tissues in comparison with non-cancerous tissues, suggesting that epigenetic modifications, such as DNA methylation and histone alteration in the coding gene, could be the underlying causes. Thus, this study may explain our predictions for the *IL-6* transcripts and support the idea that its expression level in tumor tissues is different from that in the blood of NSCLC patients.

Interestingly, this work predicted that both *IL-6* and *IL-1β* genes are positively correlated in AC and SCC subtypes. These cytokines may be linked to one another in lung cancer through autocrine and/or paracrine mechanisms (Dutkowska et al. 2021)**.** In vitro and in vivo studies illustrated that IL-1β is a key mediator of IL-6 induction via activation of the NF-κB pathway, subsequently activating the IL-6/JAK/STAT3 cascade and promoting tumor growth and aggressiveness (Tengesdal et al. 2021; Habanjar et al. 2023; Zhang and Veeramachaneni 2022). Therefore, the overall findings could suggest the significance of *IL-1β* rs16944 and *IL-6* rs1800795 as independent risk factors for the development of lung cancer. However, some limitations were found within our study, particularly because it’s a single-center study, as well as the lack of information concerning the expression levels of *IL-1β* and *IL-6* cytokines. Thus, we highly encourage ongoing research on the *IL-1β* and *IL-6* SNPs among larger populations with different ethnicities to provide more evidence for our findings. Also, further gene–gene and gene–environment studies are recommended to enhance the potential role of these cytokines in lung cancer occurrence.

## Conclusion

In conclusion, this case–control study primarily revealed the connotation between *IL-6* and *IL-1β* gene variants and NSCLC in Egyptian subjects. Our work indicated that *IL-6* gene -174C-allele carriers are significantly associated with increasing NSCLC risk, whereas the *IL-1β*-511(CT) heterozygote genotype showed a protective role for NSCLC incidence. The *IL-6*-174G > C and *IL-1β*-511C > T genetic variants could be regarded as diagnostic indicators for NSCLC. However, it is important to design further studies in other ethnic populations with larger sample sizes to support our findings. This study is considered a great insight into a deeper explanation of the mechanism of developing lung cancer, which in turn aids in improving its diagnosis and treatment.

## Supplementary Information

Below is the link to the electronic supplementary material.

Supplementary file1 (DOCX 92 KB)

Supplementary file2 (DOCX 324 KB)

Supplementary file3 (DOCX 309 KB)

Supplementary file4 (DOCX 199 KB)

## Data Availability

The corresponding author will provide the datasets upon affordable request.
