# Supplementary figures and images for: Impact of IL-6 and IL-1β Gene Variants on Non-small-cell Lung Cancer Risk in Egyptian Patients
Source: Biochem Genet. 2023 Dec 16;62(5):3367–88. doi: 10.1007/s10528-023-10596-2 (PMC11427554; doi:10.1007/s10528-023-10596-2)

| 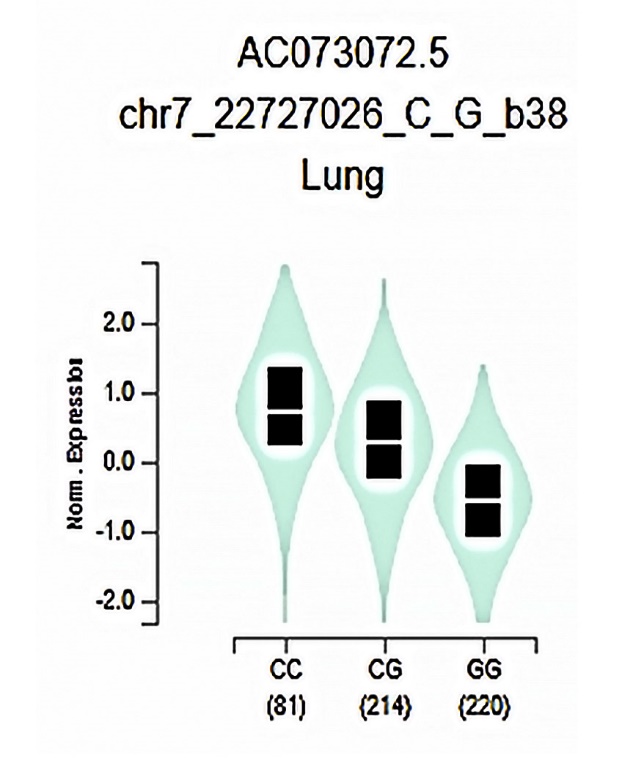  P value= 7.1e-39 |  |
| --- | --- |

**Fig. S1** Expression of *IL-6* (rs1800795) SNP in normal lung tissues (*p* = 7.1 × 10-39) using GTEx database.

Supplement: Supplementary file 1 — Supplementary file1 (DOCX 92 KB) [file 10528_2023_10596_MOESM1_ESM.docx]
