# Supplementary material for: Impact of IL-6 and IL-1β Gene Variants on Non-small-cell Lung Cancer Risk in Egyptian Patients
Source: Biochem Genet. 2023 Dec 16;62(5):3367–88. doi: 10.1007/s10528-023-10596-2 (PMC11427554; doi:10.1007/s10528-023-10596-2)
Supplement: Supplementary file 2 — Supplementary file2 (DOCX 324 KB) [file 10528_2023_10596_MOESM2_ESM.docx]

| 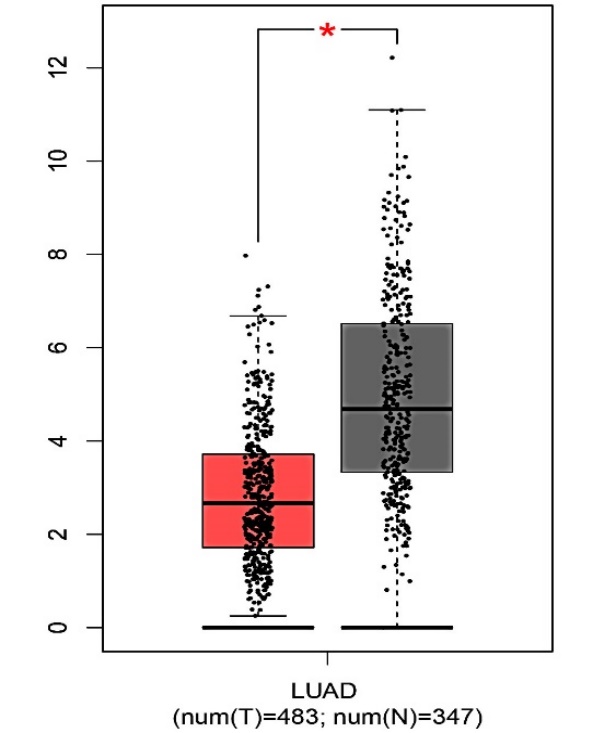**A** | 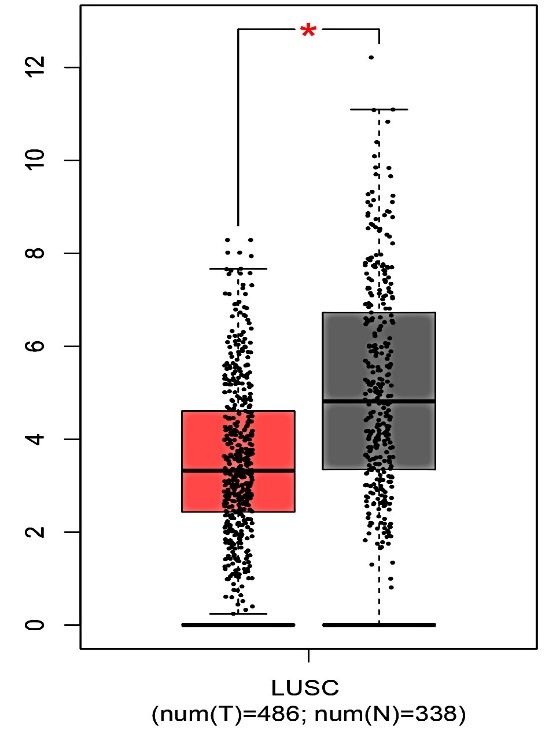**B** |
| --- | --- |
| 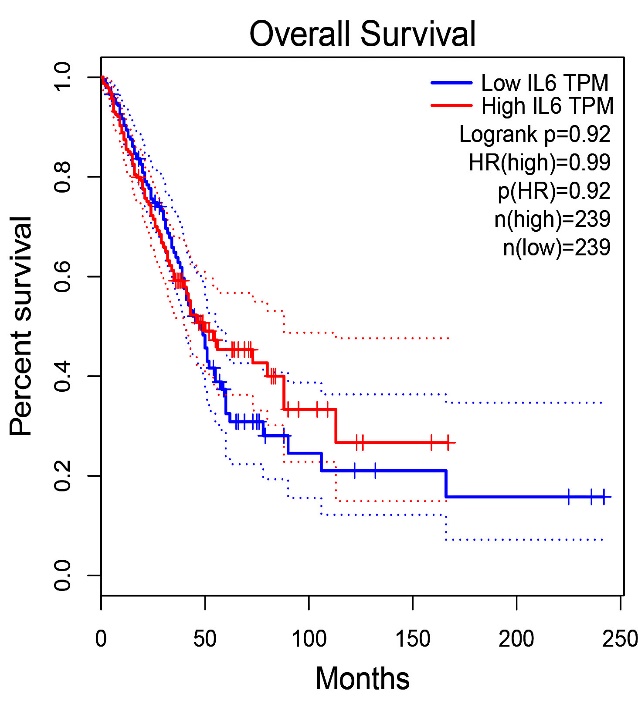**C** | 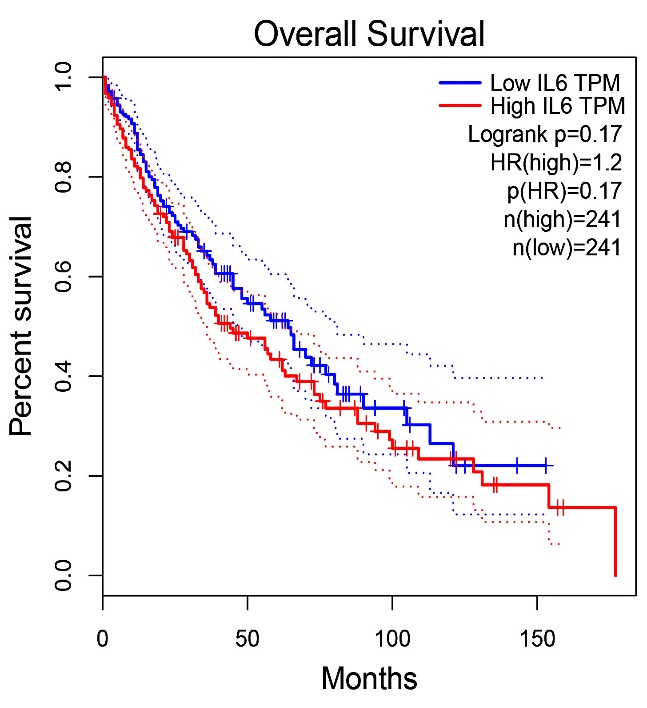  **D** |

**Fig. S2** The GEPIA database investigation of the *IL-6* gene in lung squamous cell carcinoma [LUSC] and lung adenocarcinoma [LUAD]. **(A)** The *IL-6* gene expression in para-cancerous (n=347) and LUAD tissues (n=483). **(B)** *IL-6* gene expression in para-cancerous (*n*=338) and LUSC tissues (*n*=486). The *Y*-axis displays log 2(TPM +1) (*TPM* Transcripts Per Million); the box plots display the interquartile range (IQR) and median (bar in box); and * *p* <0.01 designates statistical significance. **(C)** Relationship between LUAD prognosis and *IL-6* expression. **(D)** Relationship between LUSC prognosis and *IL-6* expression. The *Y*-axis denotes the survival rate; the red line signifies high *IL-6* expression; the blue line signifies low *IL-6* expression; a *P* value fewer than 0.05 points to statistical significance; and *HR* is the Hazard ratio.
