# Supplementary material for: Impact of IL-6 and IL-1β Gene Variants on Non-small-cell Lung Cancer Risk in Egyptian Patients
Source: Biochem Genet. 2023 Dec 16;62(5):3367–88. doi: 10.1007/s10528-023-10596-2 (PMC11427554; doi:10.1007/s10528-023-10596-2)
Supplement: Supplementary file 3 — Supplementary file3 (DOCX 309 KB) [file 10528_2023_10596_MOESM3_ESM.docx]

| 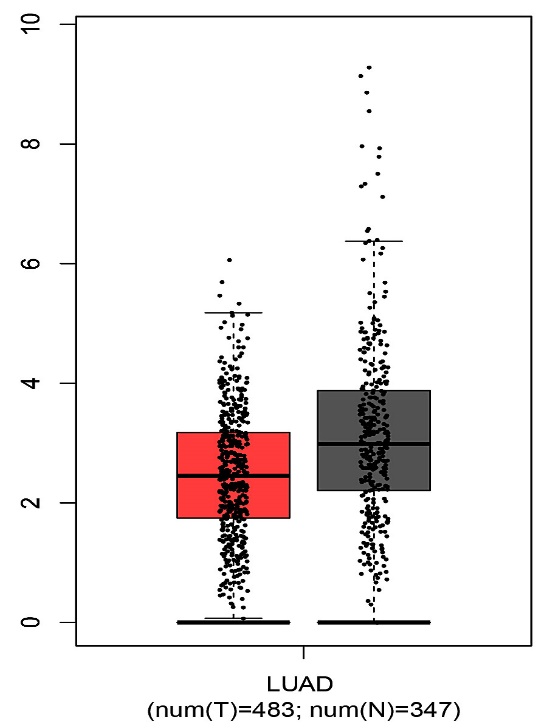**A** | 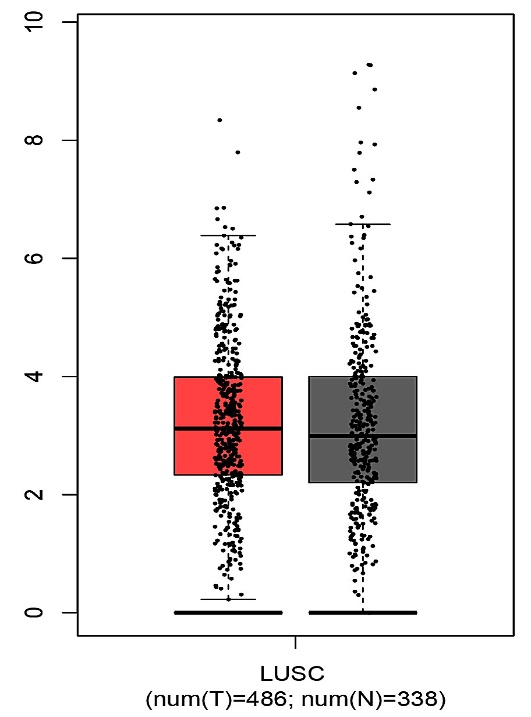**B** |
| --- | --- |
| 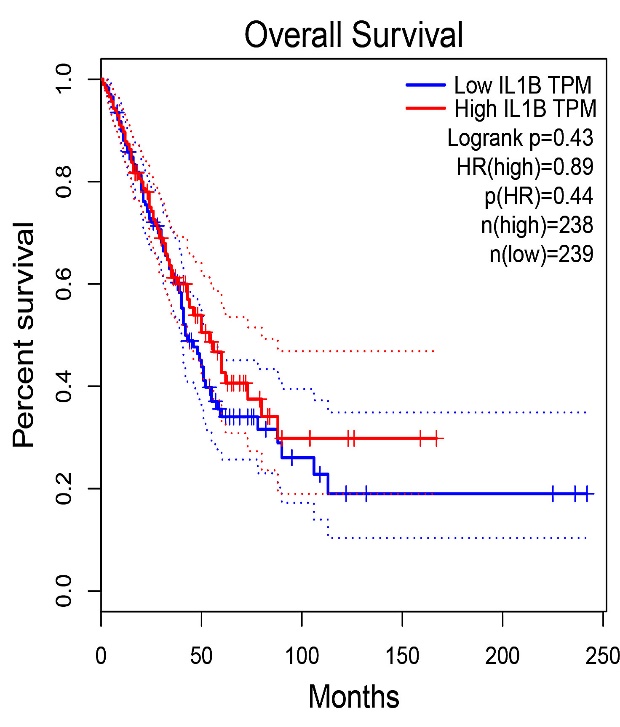**C** | 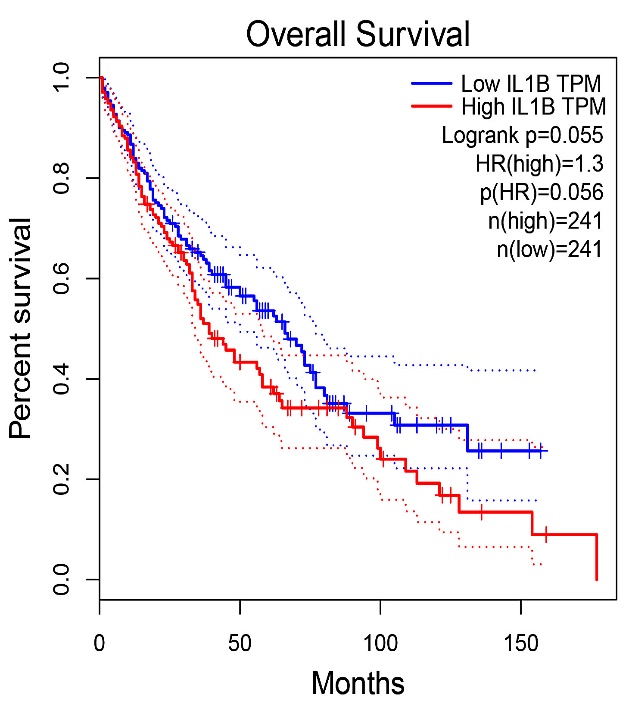  **D** |

**Fig. S3** The GEPIA database investigation of the *IL-1β* gene in lung adenocarcinoma (LUAD) and lung squamous cell carcinoma (LUSC). **(A)** *IL-1β* gene expression in LUAD (*n*=483) and para-cancerous tissues (*n*=347). **(B)** *IL-1β* expression gene in LUSC (*n*=486) and para-cancerous tissues (*n*=338). The *Y*-axis is log 2(TPM +1) (*TPM* Transcripts Per Million) and the box plots display the interquartile range (IQR) and median (bar in box). **(C)** Association between LUAD prognosis and *IL-1β* expression. **(D)** Association between LUSC prognosis and *IL-1β* expression. The *Y*-axis is survival rate; the red line signifies high *IL-1β* expression; the blue line signifies low *IL-1β* expression; a *P* value less than 0.05 specifies a significant difference; and *HR* is the Hazard ratio.
