# Supplementary material for: Impact of IL-6 and IL-1β Gene Variants on Non-small-cell Lung Cancer Risk in Egyptian Patients
Source: Biochem Genet. 2023 Dec 16;62(5):3367–88. doi: 10.1007/s10528-023-10596-2 (PMC11427554; doi:10.1007/s10528-023-10596-2)
Supplement: Supplementary file 4 — Supplementary file4 (DOCX 199 KB) [file 10528_2023_10596_MOESM4_ESM.docx]

| 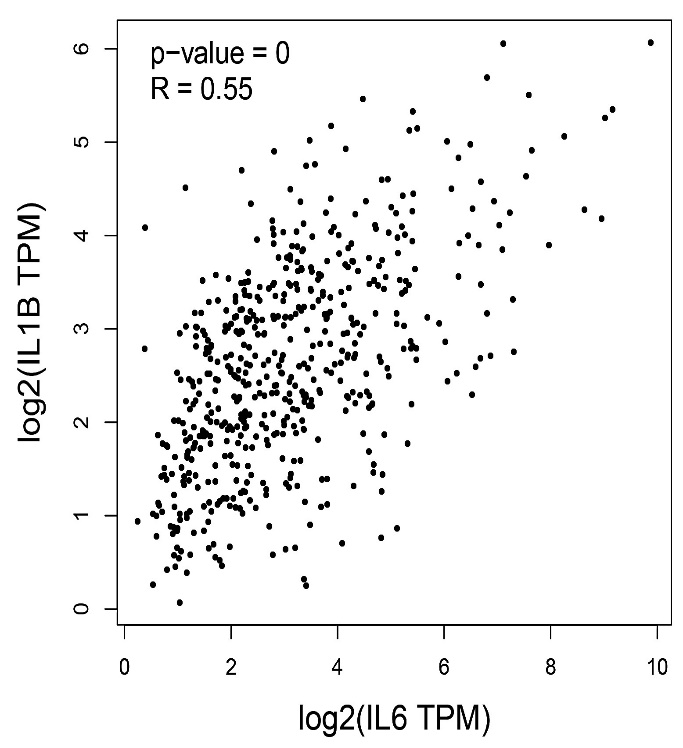**A** | 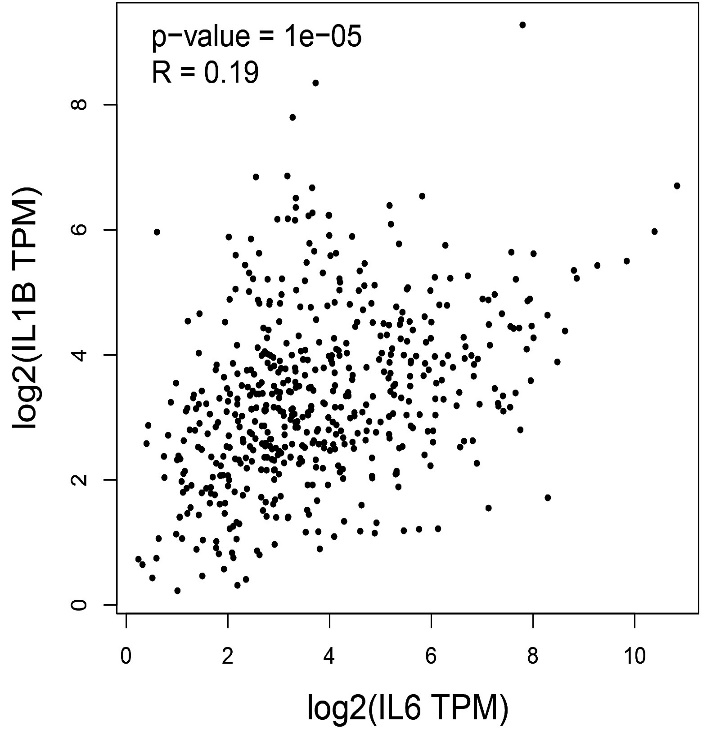**B** |
| --- | --- |

**Fig. S4** Predictive analysis of the correlation between expression of *IL-1β* and *IL-6* in lung cancer subtypes. **(A)** Correlation between genetic expressions of *IL-1β* and *IL-6* in adenocarcinoma (*p* <0.001). **(B)** Correlation between *IL-1β* and *IL-6* expressions in squamous cell carcinoma (*p* = 1.0 × 10^-5^). The *X*-axis is log 2(IL-6 TPM), and the *Y*-axis is log 2(*IL-1β* TPM). Data source: GEPIA database; TCGA expression data; Pearson method; *TPM* Transcripts Per Million.
